# Determinants of health care use among homeless individuals: evidence from the Hamburg survey of homeless individuals

**DOI:** 10.1186/s12913-021-06314-6

**Published:** 2021-04-07

**Authors:** André Hajek, Franziska Bertram, Fabian Heinrich, Victoria van Rüth, Benjamin Ondruschka, Benedikt Kretzler, Christine Schüler, Klaus Püschel, Hans-Helmut König

**Affiliations:** 1 Department of Health Economics and Health Services Research, University Medical Center Hamburg-Eppendorf, Hamburg, Germany; 2 Institute of Legal Medicine, University Medical Center Hamburg-Eppendorf, Hamburg, Germany; 3 Deutsches Rotes Kreuz Kreisverband Hamburg Altona und Mitte e.V, Hamburg, Germany

**Keywords:** Andersen’s behavioral model, Health care use, Health care utilization, Homeless, Corona-virus, COVID-19, SARS-CoV-2

## Abstract

**Background:**

To identify the determinants of health care use among homeless individuals.

**Methods:**

Data were taken from the Hamburg survey of homeless individuals (*n* = 100 individuals in the here used model, mean age 44.8 years, SD 12.5) focusing on homeless individuals in Hamburg, Germany. The number of physician visits in the past 3 months and hospitalization in the preceding 12 months were used as outcome measures. Drawing on the Andersen model of health care use as a conceptual framework, predisposing characteristics, enabling resources and need factors as well as psychosocial variables were included as correlates.

**Results:**

Negative binomial regressions showed that increased physician visits were associated with being female (IRR: 4.02 [95% CI: 1.60–10.11]), absence of chronic alcohol consume (IRR: 0.26 [95% CI: 0.12–0.57]) and lower health-related quality of life (IRR: 0.97 [95% CI: 0.96–0.98]). Furthermore, logistic regressions showed that the likelihood of hospitalization was positively associated with lower age (OR: 0.93 [95% CI: 0.89–0.98]), having health insurance (OR: 8.11 [2.11–30.80]) and lower health-related quality of life (OR: 0.97 [95% CI: 0.94–0.99]).

**Conclusions:**

Our study showed that predisposing characteristics (both age and sex), enabling resources (i.e., health insurance) and need factors in terms of health-related quality of life are main drivers of health care use among homeless individuals. This knowledge may assist in managing health care use.

## Background

In sum, about 678,000 homeless individuals lived in Germany in the year 2018 [1]. In the second largest city in Germany, Hamburg, about 6600 homeless individuals resided [2]. Moreover, the number of individuals is steadily increasing [1]. Key health-related characteristics of homeless individuals are high prevalence rates of mental disorders and infectious diseases (e.g., contracting HIV, and hepatitis B and C infections [3]). Furthermore, high prevalence rates of cardiovascular and respiratory diseases have been reported [4]. Moreover, they have a high prevalence of substance use disorders [5]. Premature death is frequent in this group [6].

Use of health care services is particularly important for the health of homeless individuals. However, only a few studies have examined the determinants of health care use among homeless individuals in German cities [7–11] mainly showing that medical services were mostly used in critical and acute situations and continued treatment rarely followed [10]. Previous international studies examining health care use among homeless individuals mostly came from North American countries such as Canada or the United States - and focused on specific topics. For instance, studies focused on oral health for homeless individuals in Vancouver [12], the use of a mobile health unit in Toronto [13], examined individuals from their entry into a homeless shelter system in New York City through the next 1.5 years [14] or investigated health care use of homeless veterans in Chicago [15]. Moreover, based on a sample of 2974 homeless persons in the United States (homeless assistance program; a nationally representative survey), Kushel et al. [16] showed that nearly 63% of individuals had one or more ambulatory care visits in the past year, about 32% of individuals visited an emergency department (ED) and more than 23% of individuals had a hospital stay. In contrast, nearly 25% were unable to receive required medical care. Particularly having health insurance was positively associated with use of ambulatory care, inpatient hospitalization, whereas it was negatively associated with barriers to required care. Furthermore, it was not associated with ED visits. A further study from Toronto, Ontario (*n* = 1165 homeless single men and women and adults in families) under a universal health insurance system showed that homeless people had markedly higher rates of ED and hospital use compared to matched controls. It should be noted that the rates were mainly driven by a subset of homeless individuals with very high use of health services [17].

A comparable recent study found that a large variety of factors (including mental health problems) predict health care use [18]. Since there is a gap in knowledge regarding health care use among homeless individuals, the aim of this study was to identify the main determinants of health care use (in terms of both physician visits and hospitalization) among homeless individuals in Hamburg, Germany.

While there may be cultural differences between Hamburg, Germany and other areas of the world, we believe that homeless populations are often characterized by traumatizing experiences, and – more broadly – similar socioeconomic and health-related factors which may be similarly associated with healthcare use in other countries.

According to the Andersen model of health care utilization [19], the determinants of health care can be distinguished into predisposing characteristics such as country of origin, sex or age, enabling resources such as perceived access and need factors such as health-related quality of life or chronic conditions.

More precisely, predisposing characteristics mainly cover social factors such as educational level or “biological factors” such as sex or chronological age. Moreover, contextual predisposing factors include, for example, cultural norms. Enabling resources cover organizational and financial factors which might be associated with health care use. For instance, individual financing factors can include wealth or income (e.g., for out-of-pocket payments). Organizational factors can include transportation or travel time. Additionally, contextual factors include, for example, density of physicians or hospitals. Moreover, it can be distinguished between evaluated need (such as chronic conditions diagnosed by physicians) and individual need (such as self-rated health) [20].

Furthermore, it has recently been proposed [21] to extend this model by adding psychosocial factors (such as loneliness, social isolation, life satisfaction, self-esteem or locus of control). Adjusting for predisposing characteristics, enabling resources and need factors, some empirical previous studies also demonstrated that psychosocial factors are important for health care use [22–26]. Since, for example, some recent studies revealed postponed access to medical services (most likely for reasons of COVID-19) [27, 28], it seems reasonable to include such a factor in our current study.

This study may assist in managing health care use and was designed to answer determinants of health care use among homeless individuals.

## Materials and methods

### Cohort description

We used data from a prospective study focusing on living conditions and health care situations of 151 homeless adults in Hamburg (“Hamburg survey of homeless individuals”), Germany. Personal interviews were conducted in night shelters, lodging houses and specialized medical practices from end of May to early June 2020 (25th May to 3rd June) using a separate room. While three individuals refused participation after initial agreement, 151 individuals took part (response rate: 98%). In sum, 100 individuals were included in the analytical sample (due to single missing values in the rest).

The visitation includes, among other things, a blood withdrawal, a physical examination, demographic information, and a questionnaire-based interview (including information about health care use). Due to problems in reading and understanding the questions, most individuals were interviewed (face-to-face) and a few individuals (without these difficulties) filled out the questionnaire independently.

All individuals provided their written and informed consent prior to their participation. The study design and content was approved by the Ethics Committee of the medical council of Hamburg (application number: PV7333).

### Dependent variables

Health care use was assessed covering (1) outpatient physician visits and (2) hospital treatment. The self-rated number of physician visits in the past 3 months was used as outcome measure. Moreover, the self-rated number of hospital visits in the preceding 12 months was assessed. Finally, data were dichotomized for this study (no hospital stay; at least one hospital stay).

### Independent variables

Drawing on the Andersen model of health care use as a conceptual framework, predisposing characteristics of the individuals, enabling resources and need factors were included as correlates. Furthermore, as recently proposed [21], we also added a very recent psychosocial factor in asking about fear of COVID-19.

As regards predisposing characteristics, we included age, sex (women or men), marital status (single vs. others (divorced; widowed; married)), educational level (according to the CASMIN classification: primary, secondary and tertiary education [29]), country of origin (Germany; neighboring country; other European country) and chronic alcohol consumption (carbohydrate-deficient transferrin (CDT > 2.5 refers to elevated) [30, 31]).

With regard to enabling resources, the potential presence of health insurance (no; yes) was included. With regard to need factors, health-related quality of life was included as need factor. It was quantified using the EQ-VAS ranging from 0 (worst) to 100 (best) [32]. Furthermore, fear of COVID-19 (from 1 = not at all to 4 = severely) was used as psychosocial factor.

### Statistical analysis

First, the analytical sample was described. Subsequently, negative binomial regressions were used (with physician visits as outcome measure) because of the nature of the data (distribution of physician visits was positively skewed) [33–35]. Hardin et al. provide further details regarding these regression models [35]. In case of hospitalization (no; yes), a logistic regression model was used. Predisposing characteristics, enabling resources and need factors were simultaneously included in this regression model. The criterion for statistical significance was set at *p* < 0.05. Analyses were performed using Stata 16.0 (StataCorp, College Station, Texas, USA).

## Results

### Sample characteristics

The analytical sample is described in Table 1. In sum, average age was 44.8 years (SD 12.5; ranging from 22 to 71 years) and most individuals were male (80%). The average number of physician visits in the past 3 months equaled 3.5 (SD 10.2 visits; from 0 to 90 visits; 44.9% of the individuals without physician visits) and 42% of the individuals reported at least one hospital visit in the past 12 months. Further details are given in Table 1.

**Table 1:** Sample characteristics (*n* = 100)

Independent variables	Mean (SD) / n (%)
Gender	
Male	80 (80.0%)
Female	20 (20.0%)
Age	44.8 (12.5)
Family status	
Single	67 (67.0%)
Widowed/Divorced/Married, living separated from spouse	33 (33.0%)
Education	
Primary education	34 (34.0%)
Secondary/tertiary education	66 (66.0%)
Country of origin	
Germany	52 (52.0%)
Neighboring country	23 (23.0%)
Other European country	25 (25.0%)
Alcohol consume	
Absence of chronic alcohol consume (CDT ≤ 2.5)	63 (63.0%)
Presence of chronic alcohol consume (CDT > 2.5)	37 (37.0%)
Health insurance	
Yes, having health insurance	69 (69.0%)
No, not having health insurance	31 (31.0%)
Fear of COVID-19 (from 1 = not at all to 4 = severely)	1.8 (1.0)
Health-related quality of life (EQ VAS, ranging from 0 (worst) to 100 (best))	75.5 (21.0)
Number of physician visits within 3 months	3.5 (10.2)
Hospitalization within 3 months	
No hospital visits	58 (58.0%)
At least one hospital visit	42 (42.0%)

### Regression analysis

The results of negative binomial regressions (with physician visits as outcome measure) are presented in Table 2. Negative binomial regressions showed that increased physician visits were associated with being female (IRR: 4.02 [95% CI: 1.60–10.11]), absence of chronic alcohol consume (IRR: 0.26 [95% CI: 0.12–0.57]) and lower health-related quality of life (IRR: 0.97 [95% CI: 0.96–0.98]). In contrast, the outcome measure was not significantly associated with age, family status, educational level and fear of COVID-19.

**Table 2:** Determinants of the frequency of physician visits in the past 3 months. Findings of multiple negative binomial regressions

Independent variables	Number of doctor visits
Gender: - Female (Ref.: Male)	4.02**
	(1.60–10.11)
Age	0.99
	(0.96–1.02)
Family status: - Widowed/Divorced/Married, living separated from spouse (Ref.: Single)	0.88
	(0.46–1.67)
Education: - Secondary/tertiary education (Ref.: Primary education)	1.59
	(0.76–3.32)
Country of origin: - Neighboring country (Germany)	2.11
	(0.79–5.64)
- Other country	0.78
	(0.41–1.49)
Alcohol consume: - Presence of chronic alcohol consume (CDT > 2.5) (Ref.: Absence of chronic alcohol consume)	0.26***
	(0.12–0.57)
Health insurance: Yes (Reference category: No health insurance)	0.70
	(0.34–1.48)
Fear of COVID-19 (from 1 = not at all to 4 = severely)	1.06
	(0.75–1.50)
Health-related quality of life (EQ-VAS, ranging from 0 (worst) to 100 (best))	0.97***
	(0.96–0.98)
Constant	11.06*
	(1.05–116.15)
Pseudo R^2^	0.10
Observations	98

Incidence rate ratios are reported. 95% confidence intervals in parentheses. *** *p* < 0.001, ** *p* < 0.01, * *p* < 0.05, + *p* < 0.10

The findings of logistic regressions (with hospitalization as outcome measure) are displayed in Table 3. Logistic regressions showed that the likelihood of hospitalization was positively associated with lower age (OR: 0.93 [95% CI: 0.89–0.98]), having health insurance (OR: 8.11 [2.11–30.80]) and lower health-related quality of life (OR: 0.97 [95% CI: 0.94–0.99]), whereas it was not significantly associated with sex, family status, educational level, country of origin, alcohol consume and fear of COVID-19.

**Table 3:** Determinants of hospitalization in the past 12 months (0 = no hospital visits; 1 = yes, at least one hospital visit). Findings of multiple logistic regressions

Independent variables	Hospitalization
Gender: - Female (Ref.: Male)	1.14
	(0.33–3.90)
Age	0.93**
	(0.89–0.98)
Family status: - Widowed/Divorced/Married, living separated from spouse (Ref.: Single)	2.42+
	(0.87–6.72)
Education: - Secondary/tertiary education (Ref.: Primary education)	0.91
	(0.31–2.70)
Country of origin: - Neighboring country (Germany)	2.68
	(0.59–12.17)
- Other country	0.93
	(0.26–3.38)
Alcohol consume: - Presence of chronic alcohol consume (CDT > 2.5) (Ref.: Absence of chronic alcohol consume)	0.82
	(0.25–2.68)
Health insurance: Yes (Reference category: No health insurance)	8.11**
	(2.13–30.80)
Fear of COVID-19 (from 1 = not at all to 4 = severely)	0.95
	(0.58–1.56)
Health-related quality of life (EQ VAS, ranging from 0 (worst) to 100 (best))	0.97**
	(0.94–0.99)
Constant	19.47
	(0.49–775.21)
Pseudo R^2^	0.22
Observations	100

Odds ratios are reported. 95% confidence intervals in parentheses. *** *p* < 0.001, ** *p* < 0.01, * *p* < 0.05, + *p* < 0.10

## Discussion

Based on recent data of homeless individuals, the authors here provide very first evidence regarding determinants of health care use in this vulnerable group exemplified for the metropolitan city Hamburg, Germany. Main results showed that increased physician visits were associated with being female, absence of chronic alcohol consume objectified by laboratory measures and lower health-related quality of life. Moreover, the likelihood of hospitalization was positively associated with lower age, having health insurance and lower health-related quality of life.

It should be noted that the number of physician visits is only slightly higher compared to the general population in Germany (average number of physician visits in the preceding 3 months: 2.8, SD 3.8) [36]. The main reason for this observation could potentially be explained by high risk lifestyle of some homeless people and being exposed to critical external influences.

Moreover, the access to healthcare services is quite good in Hamburg, Germany. For example, about two out of third homeless individuals had health insurance in our sample. While the average number of physician visits in the past 3 months equaled 4.1 visits (SD: 12.0) among homeless individuals with health insurance in our sample, it equaled 2.1 visits (SD: 3.9) among homeless individuals without health insurance. The good supply of mobile support services (e.g., rolling doctor’s office) may also explain these findings.

In contrast, the proportion of individuals with at least one hospital visit in the past year is markedly higher compared to the general population in Germany (where only 12.6% were hospitalized in the previous 12 months) [36] and most comparable to the proportion of hospital stays among the geriatric population in Germany (25.1% of the individuals reported at least one hospital stay in the past 6 months) [37]. Ignoring banal diseases on the streets and in daily life might result in exacerbation and thus, potentially more critical progressions with the necessity for hospitalization.

The positive association between frequency of physician visits and being female may be explained by the fact that homeless women may generally have a higher compliance and body awareness. They may have an increased willingness to stay healthy by using health care services – as frequently shown by studies focusing on the general population [20]. However, future research is required to clarify this association among homeless individuals in further detail. Illustrating distinct sex-associated differences in decision progress for or against physician visits were beyond the scope of this manuscript.

The link between an increased number of physician visits and the absence of chronic alcohol consume may be explained by the fact that individuals with a chronic alcohol consume may underestimate their health risks in general and their substance abuse in detail. For example, it has been demonstrated that alcohol addiction is positively associated with fatalism and feelings of invulnerability [38]. In sum, individuals with a chronic alcohol consume may therefore avoid physician visits resulting in progression of alcohol-associated organopathies.

Previous research mainly showed a link between increased needs and the number of physician visits in a variety of cohorts [39–41]. Thus, our findings with regard to the association between health-related quality of life and physician visits are well in accordance with previous research. This association can be simply explained by the fact that signs of an illness or certain symptoms were identified by the individuals and consequently the individuals are willing to check their symptoms by physicians [42]. An analogous mechanism may explain the link between health-related quality of life and hospitalization and is in line with studies using data from the general population in later decades [42, 43].

A higher likelihood of hospitalization among homeless individuals was associated with younger age in our study. We assume that younger homeless individuals may have a higher compliance regarding hospital stays compared to older individuals [44]. Moreover, younger homeless individuals may be more prone to risky behavior and alcohol poisoning or other substance use [45]. Similarly, it has been shown that conscientiousness is lower in younger age [46]. Furthermore, it has been shown that conscientiousness is associated with a lower risk of hospitalization [41]. Therefore, our findings appears plausible. However, future research in this area is required.

Additionally, there was a relevant association between having health insurance and hospitalization. This is in accordance with previous research among homeless individuals from California, US [47]. Homeless individuals not having health insurance may fear accessing hospitals (e.g., because of not being treated or being applied for payment). However, future studies (e.g., qualitative studies) are required to elucidate the underlying mechanisms exactly.

It should be noted that fear of COVID-19 was not associated with the outcome measures. Thus, use of in- and outpatient health care services (in terms of physician visits and hospitalization) is not driven by this very recent psychosocial factor. Given that recent studies showed postponed or delayed access to medical services [27, 28], partly due to fear of COVID-19, these findings are unexpected. A possible explanation for this missing association may be that fear of COVID-19 most likely only refers to the past few months, whereas hospitalization in the past 12 months was assessed. Moreover, fear of COVID-19 may be of limited importance when homeless individuals are in urgent need of care. However, future studies, e.g., based on qualitative approaches, are required to clarify why fear of COVID-19 does not drive health care use among homeless individuals and also if feelings on this pandemic phase of life changes with the second wave of infections overwhelms the world.

Several strengths and limitations of this approach are worth mentioning. First, data was used from a difficult to access and vulnerable population during the COVID-19 pandemic. However, the response rate was very high. Nevertheless, a considerable amount of missing values (mainly due to language barriers or unwillingness to answer the questions) should be noted resulting in reduction of the overall study population to a 2/3 cohort presented here. Using self-reports of health care use may introduce some recall bias. However, the recall periods have been selected in accordance with common recommendations [48]. Intentionally, the question on chronic alcohol abuse was answered with laboratory methods measuring CDT. Generally, plain and simple language was used to avoid misunderstandings during the interview [2]. We assume that our findings cannot be generalized to homeless individuals with very severe health impairments and to other regions of the world with sometimes very different national healthcare systems and supporting features. Moreover, generalizing our findings to other countries may at least be difficult, for example due to differences in access to healthcare services (in general and for homeless populations). Due to the cross-sectional nature of this study, the authors cannot dismiss the possibility that the potential direction of these associations is reversed. Moreover, additional psychosocial or personality-related determinants be included in future research [22, 40, 49]. Furthermore, other factors (e.g., other substance use disorders, health literacy, lack of transportation, cognitive functioning or perceived discrimination in health care settings) might be of importance for health care use [16, 17]. However, these factors were not included for reasons of data availability. Nevertheless, future studies should clarify the role of these factors for health care use. Additionally, it should be noted that alcohol intake was classified as predisposing characteristic [50, 51]. However, this is debatable and former studies also classified it as need factor [52, 53].

## Conclusion

This given study showed that predisposing characteristics (age or sex), enabling resources (i.e., health insurance) and need factors in terms of health-related quality of life are main drivers of health care use among homeless individuals. This knowledge may assist in managing health care use for this special but highly relevant population group.

## Data Availability

The datasets analysed during the current study are not publicly available due to ethical restrictions involving patient data but are available from the corresponding author on reasonable request.

## Abbreviations

CDT: Carbohydrate-deficient transferrin
CI: Confidence interval
COVID-19: Coronavirus disease 2019
EQ-VAS: EuroQol-Visual analogue scale
IRR: Incidence rate ratio
OR: Odds ratio
Ref: Reference category
*R*^2^: Coefficient of determination
SARS-CoV-2: Acute respiratory syndrome coronavirus 2
SD: Standard deviation
